# Nondestructive Measurement Technique for Substandard Amoxicillin Based on Thermal Approach

**DOI:** 10.1021/acsomega.4c00536

**Published:** 2024-04-18

**Authors:** Decho Surangsrirat, Onsiri Srikun, Chaksawat Sangawitayakorn, Titasmith Wannasetdecho, Mananya Puanglamjeak, Prab Birdi, Joe Kirkup, Kam Chana

**Affiliations:** † Digital Healthcare Platform Innovation Group, National Science and Technology Development Agency, Pathum Thani 12120, Thailand; ‡ Pharmaceutical Ingredient Research Group, The Government Pharmaceutical Organization, Bangkok 10400, Thailand; § Proxisense Limited, Cody Technology Park, Hampshire GU14 0LX, England; ∥ Department of Engineering Science, University of Oxford, Oxford OX1 3PJ, England

## Abstract

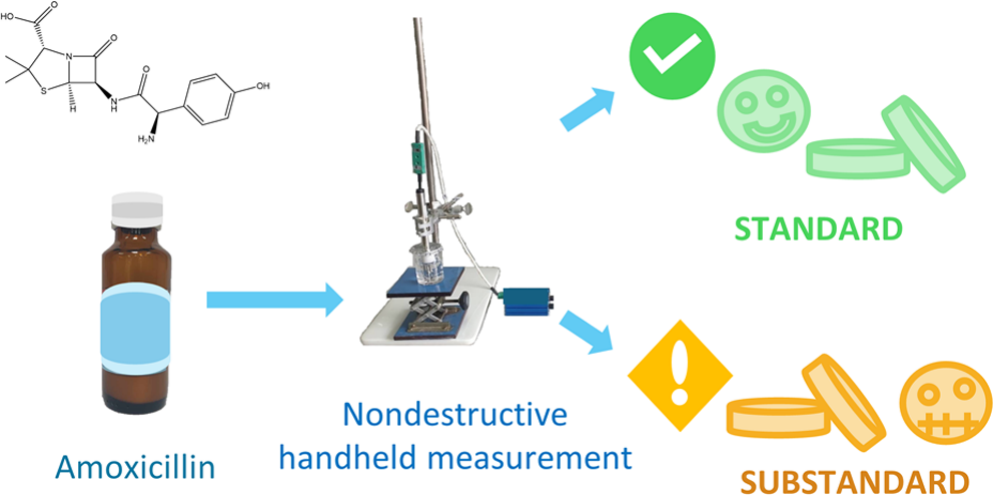

In this study, we introduce a new nondestructive measurement technique based on a thermal approach for the determination of substandard amoxicillin. The quality control of amoxicillin is critical for patient safety, and one of the essential parameters for its evaluation is the content of the active ingredient. Traditional methods for assessing amoxicillin content are defined by their time-consuming nature, reliance on skilled personnel, and frequent necessity for specific reagents. The proposed device aims to provide a rapid and low-cost alternative that can accurately measure the amoxicillin content without damaging the sample. The method validation results indicate coefficient of determination (*R*^2^) exceeding 0.99, with percent recoveries falling within the range of 98.70–103.40%. The calculated values for limit of detection and limit of quantitation were determined to be 28.11 and 85.17 mg/L, respectively. Our experiments employed amoxicillin samples with predetermined concentrations, all of which were below the standard quality. It was observed that the proposed analytical device effectively quantifies the amoxicillin content in aqueous solutions. Each measurement took no more than 10 min, underscoring the efficiency of the analysis process. The experiments were validated through independent testing at the Government Pharmaceutical Organization in Thailand and the department of engineering science in Oxford, which provides strong evidence for the effectiveness and robustness of the technique. Overall, this study demonstrates the feasibility of using a thermal approach for the nondestructive measurement of substandard amoxicillin.

## Introduction

The pharmaceutical sector has shown remarkable growth over the past decade, with revenues of $390.2 billion in 2001^1^ and reaching $1.2 trillion in 2018, which represents an increase of $100 billion from 2017 with an average growth rate of 6.3%. Projections indicate that this trend is set to continue with an expected average annual growth rate of 3 to 6% from 2014 to 2023.^2^ However, the rising profits have also attracted the attention of counterfeiters, with a surge of 102% in the production of fake medications between 2014 and 2018, according to the Pharmaceutical Security Institute (PSI).^3^ Counterfeit drugs often lack the active ingredients necessary for recovery^4^ and may contain dangerous additives, such as paint, colored dye, floor wax, boric acid, or antifreeze, in addition to incorrect amounts or concentrations. This widespread problem of drug fraud threatens the safety and well-being of patients worldwide.^5,6^

Antibiotic category accounted for up to 35% of counterfeit drug occurrences between 2014 and 2016, followed by painkillers and medications for high blood pressure, local anesthetics, diabetes, epilepsy, heart disease, and other conditions. Amoxicillin, a widely used member of the penicillin medication group, is an important antibiotic that can effectively treat a wide range of Gram-positive bacterial infections, including infections of the ear, nose, throat, lower respiratory tract, urinary tract, acute bacterial sinusitis, and skin–skin structure infections.^7^ Due to its effectiveness and versatility, amoxicillin has become a popular medication worldwide. The amoxicillin market is expected to continue to grow, with a compound annual growth rate of 3%, reaching USD 4783.5 million by 2022 and potentially increasing to USD 5812.3 million by 2028.^8^

As distinguishing between genuine and counterfeit pharmaceuticals becomes increasingly difficult based on appearance alone. The time required for inspections is inadequate for effectively curbing and stopping the production of fake drugs. For this reason, it is essential to create a tool that is fast, simple, affordable, and portable, yet able to accurately and specifically identify counterfeit products.^9^

Several methods have been used for detecting counterfeit goods. Colorimetry^10,11^ and thin-layer chromatography (TLC) offer quick, inexpensive, and user-friendly options, albeit lacking in accuracy and specificity.^12−15^ High-performance liquid chromatography (HPLC) stands out as a widely employed instrumentation method in the pharmaceutical industry, despite its costly installation and service requirements.^12^ HPLC provides the advantage of being able to integrate detectors with other techniques such as UV, MS,^16^ and electrochemical.^17,18^

Additionally, there are nondestructive methods like Raman spectrometers, which require no sample preparation, possess unique substance fingerprints, and utilize laser light wavelengths covering UV–visible to NIR ranges, enabling the analysis of organic and inorganic substances. However, they entail high maintenance costs, yield weak signals when analyzing polar substances, and necessitate expertise.^19^ X-ray diffraction & X-ray fluorescence spectroscopy,^20^ UV–vis spectroscopy, a rapid technique requiring no sample preparation with high sensitivity and selectivity, but it is cumbersome to relocate and faces limitations with complex analytes.^10,21^ Infrared spectroscopy (IR) covers the absorption range of most organic substances, facilitating the identification of functional groups in unknown compounds, with spectral signals that can be analyzed without complex calculations. Nevertheless, it is expensive, requires specific sample holders, and involves challenging sample preparation.^22^ Near-infrared spectroscopy (NIR)^23^ necessitates minimal sample preparation with high sensitivity due to photosensitive conductors, requires no sample preparation, and enables real-time analysis. However, it exhibits weak selectivity for substances with low polarity, signal overtone issues in molecules with near absorbance ranges, is costly to install and maintain, necessitates trained technicians, and presents challenges in mobility.^12^

Recent research from the University of Oxford has unveiled a new technique that employs pulsing thin film gauges to detect low concentrations of water in fuel and oil samples.^24,25^ This method quantifies the thermal effusivity of the material, which is directly correlated to the density, heat capacity, and thermal conductivity of the substance. Consequently, these can be utilized to identify various types of substances with distinct thermal characteristics, including those beyond the scope of spectroscopy. This technique is nondestructive, necessitates straightforward sample preparation, incurs minimal costs, and does not require experts to interpret results. However, there are several shortcomings, including a laborious manufacturing process and poor reliability due to the thickness of the platinum films. In our previous study, we applied this technique and developed a thermal approach sensor for the determination of water content in organic solvents such as methanol, ethanol, and isopropanol.^26^

In this study, we have improved the sensitivity of our sensor based on previous research and utilized it for the determination of liquid amoxicillin concentration. Independent testing of the developed sensors at the Government Pharmaceutical Organization (GPO) in Thailand and the Department of Engineering Science at Oxford demonstrates the efficacy and reliability of this technique. The proposed device is capable of nondestructively and accurately measuring the concentration of amoxicillin in liquid form without the need for any specific reagent, and the testing process takes only a few minutes.

## Material and Methods

### Thermal Product Theory

The thermal product theory is based on the fundamental principle of quantifying a material’s combined properties: density (ρ), specific heat capacity (*c*), and thermal conductivity (*k*). This conceptual framework is encapsulated in the equation presented below, where each variable represents one of these key properties. The theory integrates these variables to provide a comprehensive understanding of the material’s thermal behavior.^27^
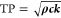


The theory asserts that the thermal product, a composite parameter, is indicative of the material’s heat dissipation characteristics, which are sensitive to changes in the material’s composition. Employing sensors designed to detect variations in heat transfer, the theory facilitates the identification of compositional changes within a material based on alterations in its thermal product. For example, in a scenario where an oil sample is contaminated, its thermal product will diverge from that of the uncontaminated oil, leading to a distinct shift in heat transfer properties. This shift is measurable; pure oil typically exhibits a thermal product in the vicinity of 500 J/m^2^ K s^0.5^, contrasting sharply with metals, where the thermal product is substantially higher, often exceeding 16,000 J/m^2^ K s^0.5^. Such precise detection of minute compositional changes via variations in the thermal product renders this theory a potent and precise instrument for identifying contamination or compositional alterations in a variety of substances. This sensitivity to subtle changes underscores the theory’s applicability and efficacy in diverse analytical contexts.

### Thermocouple Design and Development

**Figure 1:**
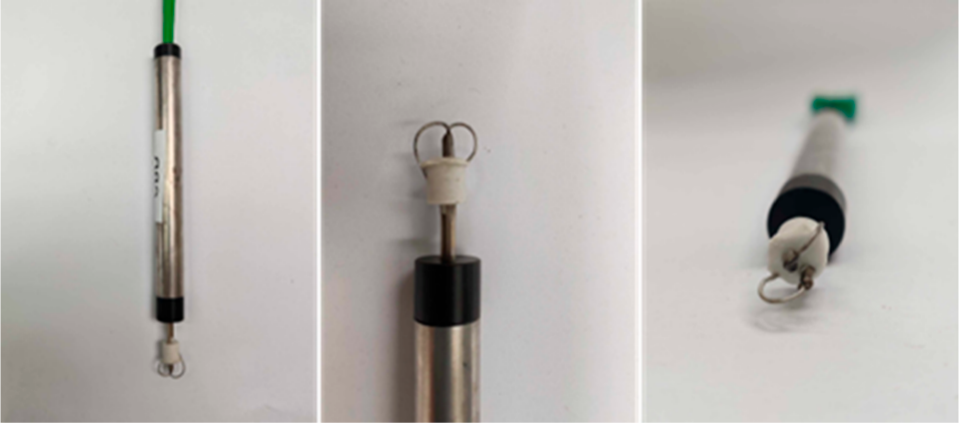
The recently developed and improved thermocouple sensor based on the work from the University of Oxford.

**Figure 2:**
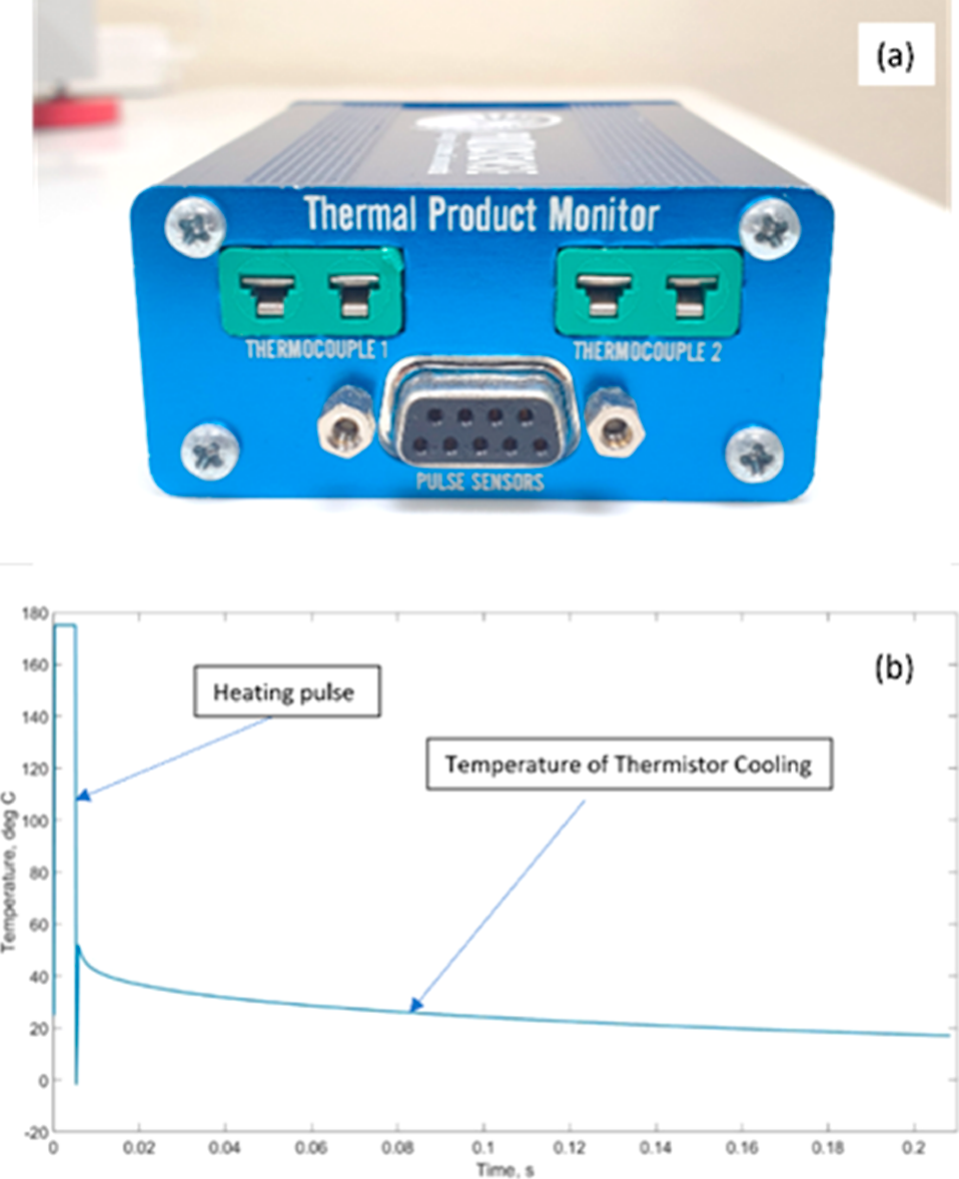
(a) Electronic box for sensor control and measurement. (b) Sample plot of a measurement pulse from the proposed device.

As depicted in Figure 1, the sensor is constructed by using K-type thermocouple wires that are electrically connected to a resistance thermistor. The flattened thermocouple wires are pressed against the thermistor’s sides and a thermally conductive paste called Loctite 9497 is applied to ensure proper thermal contact. The control and measurement of the proposed sensor are demonstrated in Figure 2a, with a sample of the measurement pulse and cooling rate illustrated in Figure 2b. The process involves transmitting a high-power pulse lasting a few milliseconds through the thermocouple wires, which heats the thermistor and the surrounding medium. Subsequently, as the pulse ceases, the thermistor cools and the temperature is measured using the thermocouple wires. The rate of cooling varies depending on the thermal product of the medium, which enables the detection of minute differences in temperature, allowing for the identification of low concentrations of impurities. This technique is more cost-effective, robust, and less noisy compared to the previous approach, as the heating and measurement steps are separated.

### Experimental Setup

Deionized water was prepared in-house using a Pacific TII 40 (UV). Organic solvents that we used in this study were purchased from Honeywell, Merck, or Colosol. For the experiment, a series of samples were chosen from two locally recognized Thai brands of amoxicillin: “Coamox” and “Siamox”. This selection allowed for a comprehensive analysis across multiple specimens from each brand.

The testing of amoxicillin was divided into low concentration and substandard ranges. The sensitivity and specificity of the detection method differ notably across the various concentration levels. Accurate measurement of lower concentrations requires highly sensitive detection techniques, while the assessment of higher concentrations may necessitate methodological adjustments to counteract potential saturation effects on the detection system. This bifurcation facilitates a comprehensive understanding of our proposed method’s characteristics and accuracy across a spectrum of concentrations.

Furthermore, the separation into two concentration ranges enables the determination of the analytical device’s linear response range, ensuring precise and reliable measurements across a varied concentration spectrum. It also assists in identifying and understanding potential nonlinear behaviors and interactions that are particularly pertinent at higher concentrations, a crucial aspect for accurate data interpretation. This approach also mirrors real-world conditions where amoxicillin levels exhibit significant variability.

**Figure 3:**
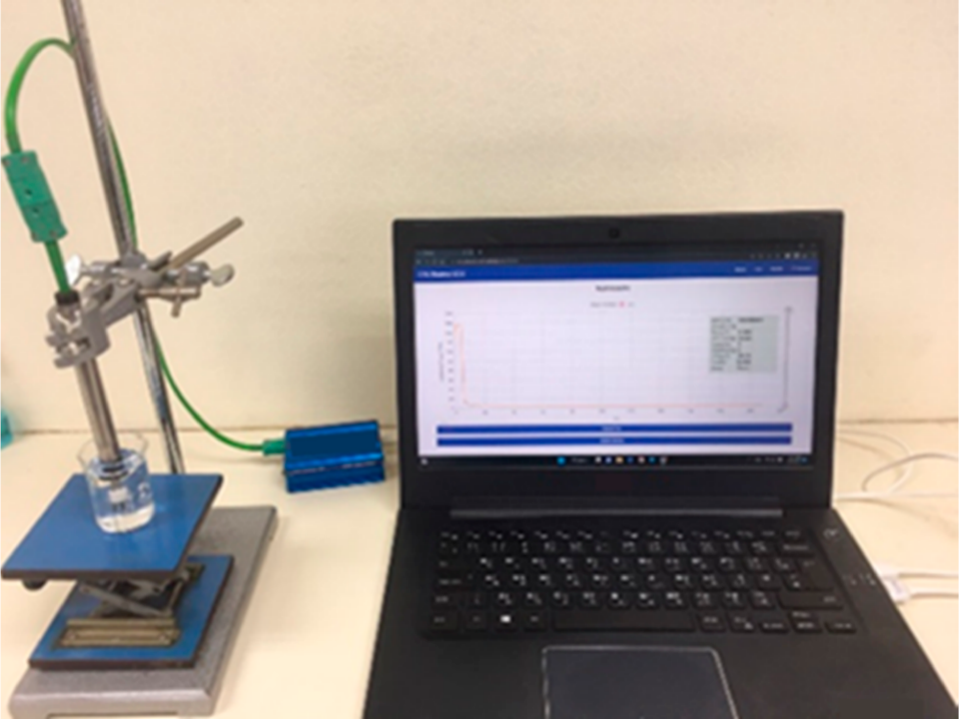
Testing setup for the proposed device. The measurement was performed by fully submerging the sensing element in the sample.

Concentration of Amoxycillin shown on the label is 50,000 mg/L; with these considerations in mind, we opted to conduct amoxicillin concentration tests within two defined ranges for this experiment: a low concentration range at 0, 10, 20, 40, and 80 mg/L and a substandard range at 600, 1200, 1800, 2400, and 3000 mg/L. This structured approach ensures a thorough and nuanced evaluation of the analytical method, catering to the diverse scenarios encountered in amoxicillin analysis. All the experiments were performed in triplicates. Figure 3 illustrates the setup and the sample testing process. The measurement was performed by fully submerging the sensing element of the sensor into the sample for approximately 10 min.

### Validation of Method

The developed method was validated for linearity, accuracy, limit of detection (LOD), and limit of quantification (LOQ) in accordance with ICH guideline for validation of analytical procedures Q2 (R1). Stock solutions of amoxicillin trihydrate were prepared as standard solutions in deionized water at a concentration of 1000 mg/L for the proposed device and a concentration of 500 mg/L for the UV–vis gold standard.

### Method Validation—Linearity

Six standard solutions of amoxicillin trihydrate were prepared at concentrations of 100, 150, 200, 250, 300, and 350 mg/L in deionized water. Each of these standard solutions underwent triplicate analysis. A calibration curve was constructed by plotting the delta temperature against the concentration of amoxicillin trihydrate. Linearity was evaluated by determining the slope, *y*-intercept, and coefficient of determination (*R*^2^) using least squared regression.

### Method Validation—LOD and LOQ

The linear regression equation of the calibration curve was utilized to establish the LOD and LOQ. These were derived from the standard deviation of the *y*-intercept and the slope (s), using formulas of 3.3SD/s for the LOD and 10SD/s for the LOQ.

### Method Validation—Accuracy

An accuracy of the method was assessed at three distinct concentrations. Samples were prepared by spiking standard solutions with deionized water to give concentrations of 150, 200, and 250 mg/L. Each concentration underwent analysis in triplicate (*n* = 3). Accuracy is represented as the percentage recovery of added amoxicillin trihydrate.

### Experimental Sample Preparation

Two Thai Amoxicillin brands “Coamox” and “Siamox” were pipetted 5 mL into 50 mL volumetric flask and dilute with deionized water. The solution was filtered using a 0.45 μm Millipore filter, and 8 mL of the filtrate was added into a 100 mL volumetric flask. Deionized water was then added to complete the volume. All equipment used for sample preparation in this experiment undergoes calibration and certification in accordance with the quality control manual of the Thai GPO.

### Sample Accuracy Evaluation

For the comparison and evaluation of the proposed device, ultraviolet–visible spectrometry (UV–vis) was employed as the gold standard based on the established reliability and accuracy of UV–vis in similar analytical research. To ensure the repeatability and consistency of our method, we undertook three separate measurements. This approach not only reinforced the validity of the results but also provided an opportunity to assess the precision of our device in comparison to UV–vis spectrometry.

## Results

In this experiment, our proposed device utilized the principle of thermal product theory to analyze amoxicillin concentrations. This approach involves detecting variations in the thermal behavior of solutions at different concentrations by quantifying a material’s combined properties: density (ρ), specific heat capacity (*c*), and thermal conductivity (*k*). The developed method was validated for linearity, accuracy, LOD and LOD in accordance with ICH guideline for validation of analytical procedures Q2 (R1). We tested the device in the sample from two groups of amoxicillin concentrations: low and substandard concentrations. The low concentration levels were set at 0, 10, 20, 40, and 80 mg/L, while the substandard included 600, 1200, 1800, 2400, and 3000 mg/L. This broad spectrum allowed for a comprehensive assessment of the device’s performance under various conditions. To benchmark the accuracy of our thermal product theory-based device, we compared its results with those obtained from UV–vis.

This comparison was facilitated by calculating the linearity determination coefficient. Each sample underwent three separate measurements.

### Method Validation

**Figure 4:**
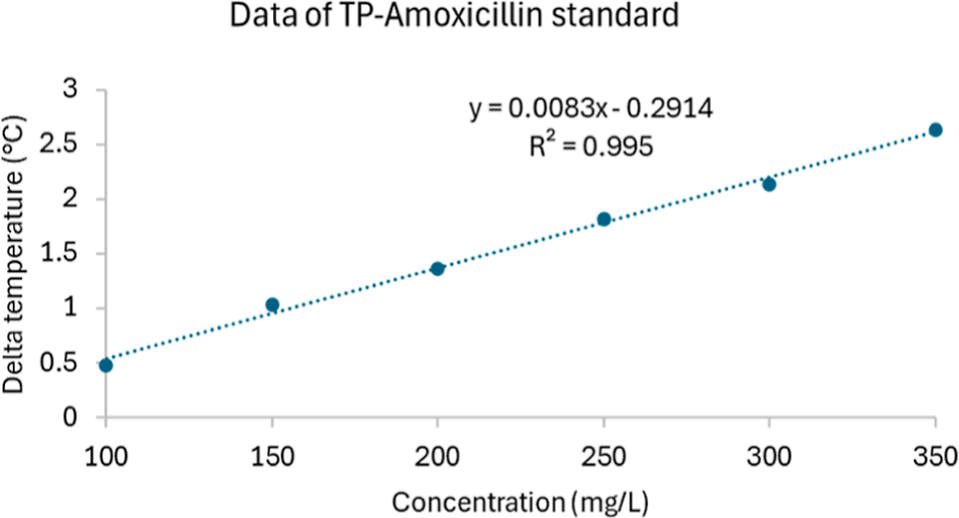
Linearity of the proposed device for amoxicillin standard in deionized water. The *x* axis is the concentration of amoxicillin, and the *y* axis is the Delta temperature measured from the proposed device.

The calibration curves exhibited linearity within the tested concentration ranges, as shown in Figure 4. The coefficient of determination (*R*^2^) exceeded 0.99, indicating a strong correlation and good linearity of the method. Consequently, the calculated values for LOD and LOQ were 28.11 and 85.17 mg/L, respectively. For the accuracy assessment, the percentage recovery of the spiked sample was calculated using the formula % recovery = [((*S*_1_–*S*_2_)/*S*_0_) × 100], where *S*_0_ is the concentration of the spiked standard amoxicillin solution, *S*_1_ is the concentration of the spiked sample, and *S*_2_ is the concentration of the unspiked sample. As described in Table 1, percent recoveries fell within the range of 98.70–103.40% across all concentrations (*n* = 3), indicating a good accuracy of the method.

**Table 1:** Accuracy of the Proposed Device Using Standard Solution with Three Different Concentrations of Amoxicillin Trihydrate^a^

sample	concentration added (mg/L)	concentration measured (mg/L)	recovery (%)
150 mg/L amoxicillin standard in DI water	150.00	152.46	101.64
200 mg/L amoxicillin standard in DI water	200.00	197.41	98.70
250 mg/L amoxicillin standard in DI water	250.00	258.49	103.40

a Samples were prepared by spiking standard solutions with deionized water to give concentrations of 150, 200, and 250 mg/L. Each concentration underwent analysis in triplicate (*n* = 3).

### Low Concentration Amoxicillin Samples

**Figure 5:**
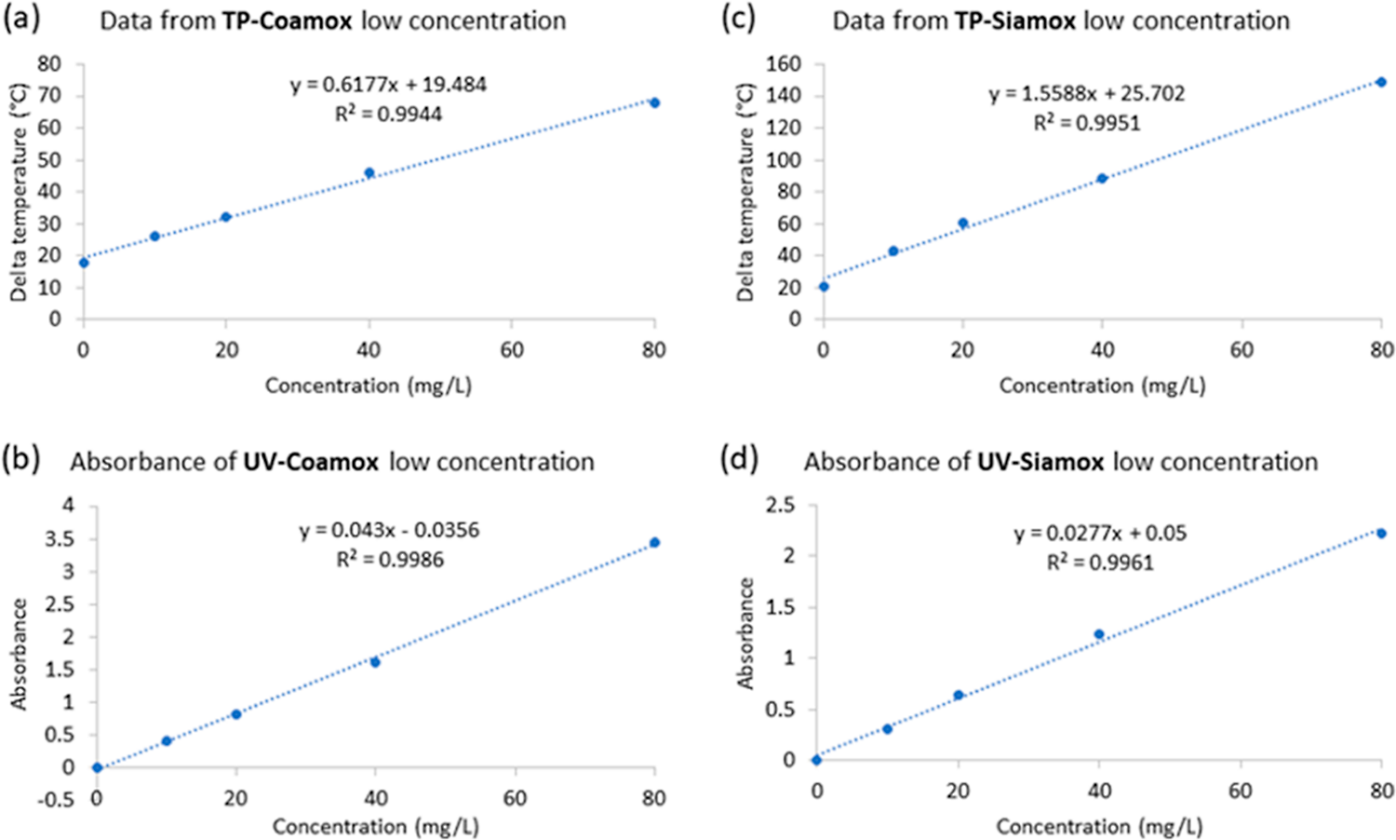
Comparative analysis of the measurement of the substandard amoxicillin samples (0–80 mg/L) using the proposed device and UV–vis for “Coamox” and “Siamox”. The top graphs (a) and (c) illustrate the relationship between the concentration of amoxicillin (mg/L) and the corresponding delta temperature as measured by the proposed device. The bottom graphs (b) and (d) show the absorbance measured by UV–visible spectrometry. Both methods demonstrate high linearity in measuring low-concentration amoxicillin, as evidenced by their respective *R*^2^ values, which are indicative of the proposed device’s reliability in comparison to the established UV–vis method.

The analysis of low concentration amoxicillin (0–80 mg/L) using the proposed thermal product device and UV–visible spectrometry produced highly linear results for both “Coamox” and “Siamox”. For “Coamox”, the device demonstrated a delta temperature with an *R*^2^ of 0.9944 as shown in Figure 5a, indicating a strong linear correlation between concentration and thermal response. “Siamox” samples yielded an even higher linearity with an *R*^2^ of 0.9951 presented in Figure 5c. Correspondingly, UV–visible spectrometry readings for “Coamox” in Figure 5b exhibited a near-perfect linear relationship with an *R*^2^ of 0.9986, while “Siamox” displayed an *R*^2^ of 0.9961 in Figure 5d. These results suggest that both the proposed device and UV–vis spectrometry are highly effective for analyzing low concentrations of amoxicillin, providing a strong indication of their reliability for precise measurement.

### Substandard Amoxicillin Samples

**Figure 6:**
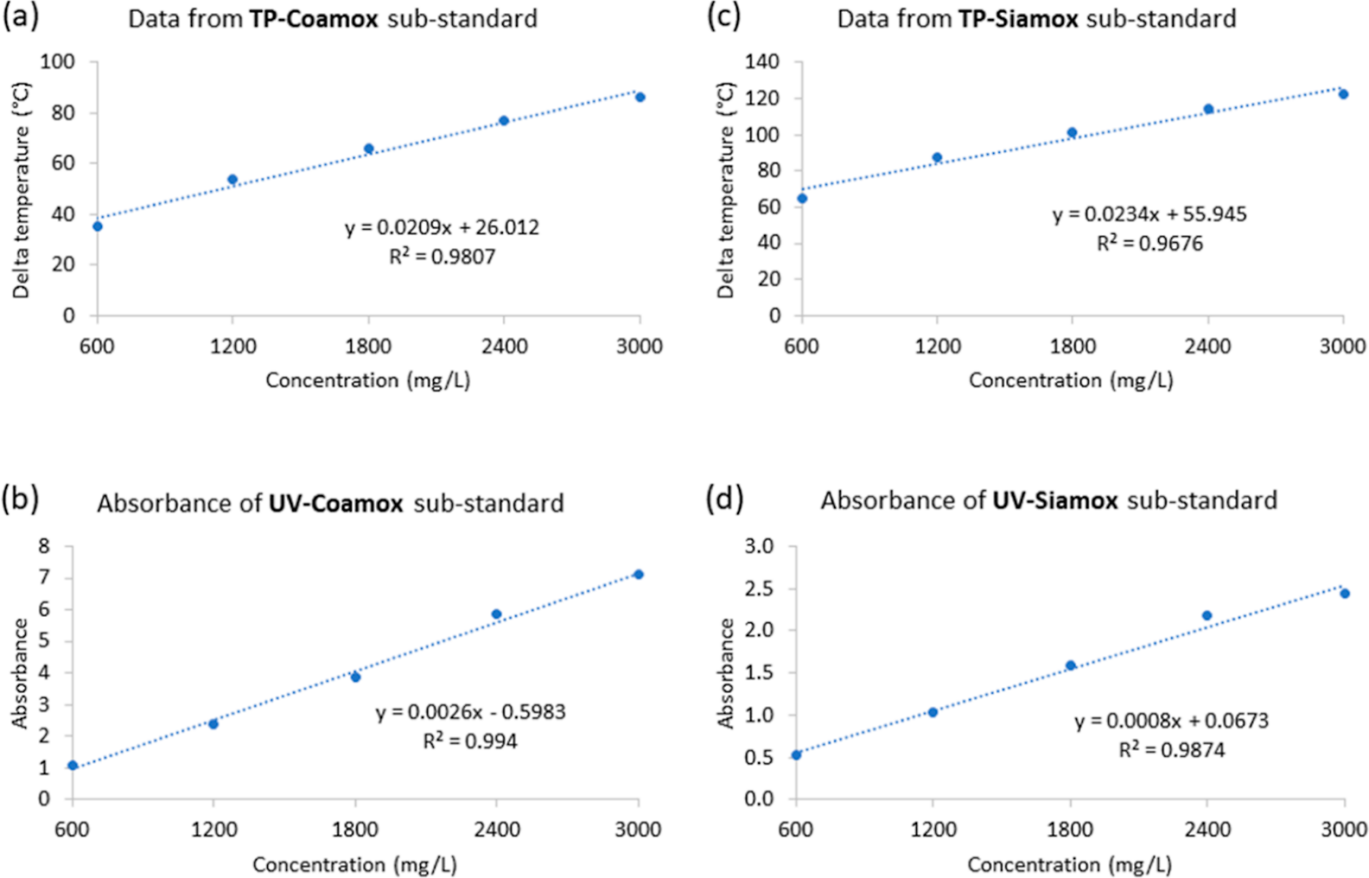
Comparative analysis of the measurement of the substandard amoxicillin samples (600–3000 mg/L) using the proposed device and UV–vis for “Coamox” and “Siamox”. The top graphs (a) and (c) illustrate the relationship between the concentration of amoxicillin (mg/L) and the corresponding delta temperature as measured by the proposed device. The bottom graphs (b) and (d) show the absorbance measured by UV–visible spectrometry. Both methods demonstrate high linearity in measuring substandard amoxicillin, as evidenced by their respective *R*^2^ values, which are indicative of the proposed device’s reliability in comparison to the established UV–vis method.

At a higher level of concentration but still below the necessary standard for amoxicillin, hence categorized as the substandard concentrations group (600–3000 mg/L), the proposed device and UV–visible spectrometry continued to show robust linearity in their respective measurements. The thermal product device recorded an *R*^2^ of 0.9807 for “Coamox”, and an *R*^2^ of 0.9807 for “Siamox” as shown in Figure 6a,c respectively, affirming the method’s effectiveness across a broader concentration range. UV–visible spectrometry readings were similarly linear for “Coamox” with an *R*^2^ of 0.9940 and for “Siamox” with an *R*^2^ of 0.9676 (Figure 6b,d.) These high *R*^2^ values confirm that the linearity of the response is maintained at a substandard, underscoring the potential of the thermal product device as a reliable method for amoxicillin quantification in a wide range of concentrations.

## Discussion

The method validation results for the proposed device demonstrate acceptable linearity, LOD, LOQ, and accuracy. The experimental data from the sample measurements indicate that the proposed device shows a strong linear relationship for the measurement of the concentration of amoxicillin for the two brands “Coamox” and “Siamox”. The consistency in the linearity of the device’s response to both low and substandard amoxicillin suggests that the proposed method is reliable across a wide dynamic range. The slightly higher *R*^2^ values observed with the UV–visible spectrometry confirms its status as a gold standard; however, the comparable results from the thermal product-based device imply its potential as a viable alternative for amoxicillin measurement. The high degree of correlation between the device measurements and UV–visible spectrometry results across both brands and concentration ranges strengthens the argument for adopting thermal product-based measurements in settings where traditional spectrometry methods may be less accessible or practical.

The device offers multiple advantages that bolster its utility in diverse environments. Each measurement was completed in 10 min during our experiments, with potential for further time reduction without compromising accuracy. Its portable design enables on-site analysis, offering immediate results. This feature, combined with the device’s nondestructive approach to measurement, is especially beneficial in locations lacking traditional lab infrastructure, thus supporting prompt quality control and decision-making. Consequently, the device’s efficiency and on-demand diagnostic capability cater well to the pharmaceutical industry’s need for quick and precise analytical instruments.

There are also limitations of our proposed method. First, the device parameters require fine-tuning and setup tailored to specific measurements. Once configured, the device exhibits the ability to accurately detect changes or variations within that setup. Customizing the device becomes essential when it is used with various substances or for different purposes, necessitating adjustments to the parameters accordingly. Second, since the device operates by measuring parameters linked to the thermal properties of substances, variations in environmental temperature could affect the accuracy of the readings, especially in locations where temperatures may fluctuate during testing. In addition to controlling the environmental temperature during measurements, employing improved temperature offset techniques could enhance accuracy.

## Conclusions

Antibiotic counterfeits are becoming increasingly difficult to detect by only appearance, and if there are no instruments available to quickly block this corruption, it could have unavoidable negative consequences. The standard methods like colorimetry and TLC are inexpensive and quick but lack accuracy and specificity. Although nondestructive with high sensitivity and specificity like HPLC, Raman, X-ray, UV–vis, IR, and NIR exist, they are all expensive, require a trained technician, specific reagents, take a long time to measure, and difficult to move around. Thus, they are not suitable for both online and in-line applications.

We establish the effectiveness of the proposed device for detecting various substandard concentrations of amoxicillin in water ranging from 0 to 3000 mg/L. Method validation results reveal a coefficient of determination (*R*^2^) exceeding 0.99, with percent recoveries within the range of 98.70–103.40%. The calculated values for LOD and LOQ are determined to be 28.11 and 85.17 mg/L, respectively. Additionally, sample measurements demonstrate a strong correlation between the device’s readings and UV–visible spectrometry. Further experiments could explore the potential of utilizing a thermal approach for the nondestructive measurement of substandard amoxicillin.

Moreover, the ability of the thermal product-based device to maintain linearity at higher concentrations is particularly noteworthy, as it suggests the device’s capability to handle a broad spectrum of analytical demands without compromising accuracy. This could have significant implications for rapid, on-site testing applications, offering a more efficient and cost-effective solution for the quality control of pharmaceuticals. The aforementioned findings may also open the door for the future development of online and in-line measurements to support process analytical work.
